# Radiation and Energetic Analysis of Nanofluid Based Volumetric Absorbers for Concentrated Solar Power

**DOI:** 10.3390/nano8100838

**Published:** 2018-10-16

**Authors:** Jan Rudolf Eggers, Eckart Matthias Lange, Stephan Kabelac

**Affiliations:** Institute for Thermodynamics, Leibniz University Hannover, Callinstraße 36, D-30167 Hannover, Germany; eggers.jr@googlemail.com (J.R.E.); kabelac@ift.uni-hannover.de (S.K.)

**Keywords:** nanofluid, solar absorption, energy conversion, temperature distribution, spectral penetration depth, scattering, anisotropy

## Abstract

Recently, several publications gave attention to nanofluid based solar absorber systems in which the solar radiation energy is directly absorbed in the volume of the fluid. This idea could provide advantages over conventionally used surface absorbers regarding the optical and thermal efficiency. For the evaluation of this concept, a numerical approach is introduced and validated in this contribution. The results show that the optical efficiency of a volumetric absorber strongly depends on the scattering behavior of the nanofluid and can reach competitive values only if the particle size distribution is narrow and small. If this is achieved, the surface temperature and therefore the heat loss can be lowered significantly. Furthermore, the surface absorber requires very high Reynolds numbers to transfer the absorbed energy into the working fluid and avoid overheating of the absorber tube. This demand of pumping power can be reduced significantly using the concept of volumetric absorption.

## 1. Introduction

Judging from the increasing number of publications, nanofluids still excite the scientific world about two decades after their first appearance in the literature [1]. The intriguing idea behind the concept of nanofluids is the possibility to change a heat carrier’s thermophysical properties by addition of small amounts of solid nanoparticles. Articles have been published describing the effective density [2,3], specific heat [4,5], thermal conductivity [6,7,8] and viscosity of nanofluids [9,10], with especially the latter two still being a matter of lively discussion.

Besides the questions of fundamental research, nanofluids are also suggested to be used in a variety of technical applications [11,12,13,14] with solar absorption being one of them. Presently, solar thermal power plants focus radiation on an opaque absorber tube in which a working fluid of a thermodynamic cycle is heated. Such a system design, even developed to a very sophisticated level, involves in principle some mechanisms causing increased thermal losses and entropy generation [15]. The reason is that energy is absorbed at the outer surface of the absorber tube and subsequently conducted to the inner tube surface, and then transferred into the working fluid by convection. The second law of thermodynamics yields that highest temperatures occur at the absorber surface. This absorber surface temperature is the key parameter for thermal losses of an absorber system. Burkholder and Kutscher [16] quantified the thermal losses as a function of the surface temperature for the SCHOTT PTR70 solar thermal absorber.

**Figure 1:**
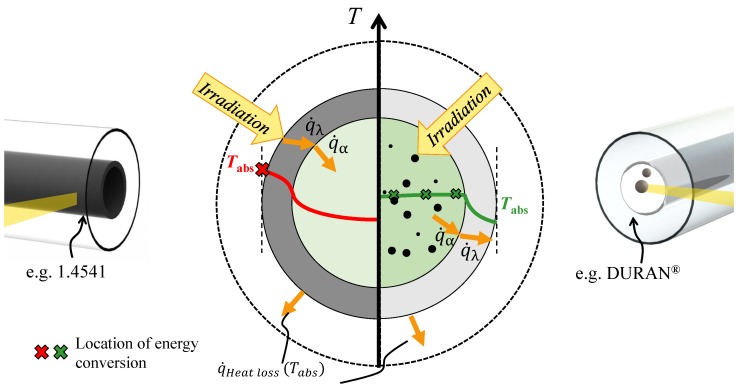
The figure illustrates that the location of radiation absorption is relocated from the outer surface into the absorber fluid. This results in a different temperature distribution and lower surface temperatures.

An idea to overcome these issues was already suggested decades ago, e.g., by Minardi and Chuang [17]. The authors discussed a mixture of Prestone II antifreeze with either India ink or Aquadag as an ingredient to modify the optical properties of the liquid. The solar irradiation passed the transparent tube and was directly absorbed by the *Black Liquid*. Minardi and Chuang concluded that, by further modifications of the flow pattern, the effect can be used to generate the hottest spot inside the flow, far away from the walls. In Figure 1, these two systems are compared, the classical surface absorber (left) and the volumetric absorber (right). The locations where radiation is absorbed are shown schematically. It can be seen from the drafted temperature curves that a lower surface temperature for the volumetric system is expected which will result in decreased thermal losses.

Similar concepts were discussed after nanofluids emerged in the scientific community (e.g., [18,19,20]), as nanofluids can show strong absorption behavior, especially in the solar spectrum (e.g., [21,22,23,24]). A slightly different concept is to use the strong absorption capability of small particles dispersed in a gas flow, as was already suggested by Hunt [25]. Recently, de Risi et al. [26] performed calculations with 0.3 vol. % CuO and Ni particles in an air stream and achieved a solar to thermal efficiency of 62.5%.

Li et al. [27] showed an experimental setup of a concentrated solar thermal collector with multi-walled carbon nanotubes in deionized water (DI) as base fluid. A fresnel lens and a mirror system focus the solar irradiation on a transparent absorber tube in which the nanofluid is heated and compared to a CU coated absorber tube. In their experiments, the authors found a 12% higher efficiency of the surface absorber compared to the new concept. However, the authors concluded that reflections on the glass surface and thermal losses explain this difference. They concluded that an optimized absorber design, including a selective coating, will yield a higher absorption efficiency of the volumetric absorber.

Xu et al. [28] simulated the temperature distribution in a parabolic trough system that absorbs radiation volumetrically in a CuO nanofluid with synthetic oil as base fluid. They found that the volumetric absorber shows higher thermal efficiencies at low inlet temperatures due to more uniform temperature fields but did not consider the entire optical processes during the absorption.

In this contribution, the focus is on the discussion of the radiation absorption and the resulting temperature field in the tube wall and absorber fluid and its impact on the thermal losses for both absorber types. For the discussion, numerical simulations are conducted for both concepts, where Ag-H${}_{2}$O nanofluid was chosen for the analysis of the volumetric absorber, as it has been shown before that it is suitable for this application [29]. It must however be stated that the variation of particle material as well as the base fluid properties have a great impact on the nanofluid absorber performance and are further possibilities for optimization. To gain more understanding of such a system, prior simulations (e.g., [28]) are widened by considering the spectral distribution of both, the solar spectrum and the absorption and scattering spectra as well as the particle size distribution (PSD) of the nanofluid.

## 2. Numerical Results and Discussion

The following section summarizes the key findings of the calculations. Many calculations were performed to evaluate the influence of the particle volume fraction, size distribution, flow velocity, absorber geometry and spectral differences in the radiation field. Data are given for the most important aspects of the results.

### 2.1. Discussion of the Radiation Field

An important issue of nanofluids is that especially the optical properties can be modified by alternation of the volumetric particle fraction. This adaption can be a measure to move the hotspots away from the absorber surface and hence reduce thermal losses. In fact, this procedure is quite sophisticated and is discussed in this section.

Radiation distribution within the volumetric absorber is a function of wavelength, spectral dependent absorption and scattering coefficient, absorber geometry and particle size distribution and volume fraction $f_{V}$ defined as

(1) $$f_{V}=\frac{V_{particle}}{V_{particle}+V_{basefluid}}.$$

**Figure 2:**
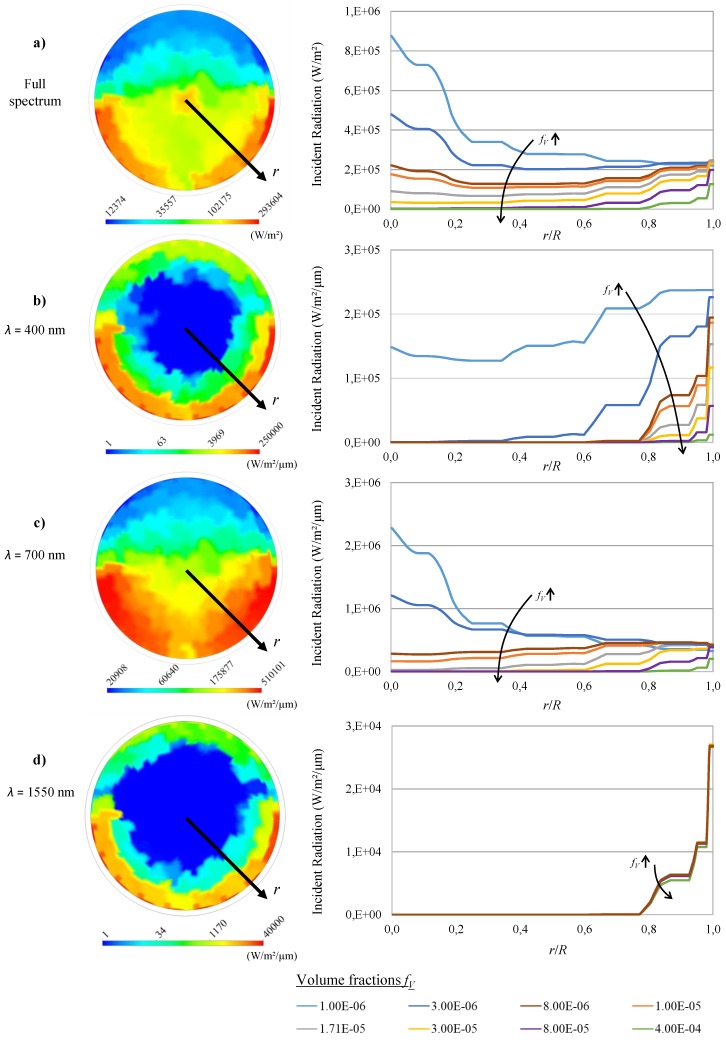
Distribution of radiation intensity in the absorber cross-sectional area for: (**a**) the solar spectrum; (**b**) λ = 400 nm; (**c**) λ = 700 nm; and (**d**) λ = 1550 nm for different particle volume fractions $f_{V}$.

To optimize the temperature field, it is crucial to gain knowledge about the radiation distribution. To discuss the impact of particle volume fraction, four representative radiative situations (compare Figure 10) were selected, as can be seen in Figure 2.

Figure 2a shows the radiation distribution for the solar spectrum. The left part shows the spatial distribution in the cross-sectional area of the volumetric absorber tube for a particle volume fraction of $f_{V}$ = 1 $\times \;10^{-5}$. The image emphasizes the earlier statement that the radiation distribution, being influenced by several factors, can hardly be predicted except doing numerical calculation. A decay of the radiation with growing distance from the walls in the area of high incident radiation at lower sides of the absorber can be seen, as it is expected from the Beer–Lambert law [30]. This trend inverts at some point in the center when more radiation is focused on a smaller area. In fact, the radiation intensity inclines to a maximum in the center, which is the desired effect when talking about thermal trapping (note the log-scale). Furthermore, the influence of lower incident radiation rates can be seen clearly at the upper surface, where there is no concentrated solar radiation, as well as at the bottom of the absorber, where the shadow of the absorber tube has an effect (compare Figure 9). To analyze the impact of particle volume fraction, the radiation intensity is exemplified along the dimensionless radius (black line in the left part of Figure 2) in a downwards 45${}^{\circ }$ angle at the right side of the Figure for different volume fractions ranging from $f_{V}$ = 1 $\times \;10^{-6}$ to $f_{V}$ = 4 $\times \;10^{-4}$. A strong dependency on $f_{V}$ can be detected: Low particle loads cause an increase of radiation intensity towards the center of the absorber, but raises the risk of allowing photons to be transmitted through the absorber. High values of $f_{V}$ on the other hand cause a steep decay of radiation intensity at the absorber wall so that it tends to zero towards the center.

As mentioned above, a spectral analysis is needed for a more detailed evaluation of the absorber. Hence, the radiation intensity for a wavelength of λ = 400 nm is shown in Figure 2b. This case was selected because the wavelength matches the absorption maximum of Ag nanoparticles, whereas the base fluid H${}_{2}$O is highly transmissive (compare Figure 10). As can be seen from the left part of the figure for a moderate volume fraction of $f_{V}$ = 1 $\times \;10^{-5}$, most of the short wave radiation is absorbed close to the absorber wall (note the log-scale). The influence of the particle volume fraction is shown along the black line of dimensionless radius at the right side of the figure again. It can be seen that, with decreasing volume fraction, more short wave radiation is transmitted towards the center of the absorber. A high volume fraction on the other hand would increasingly react like a surface type absorber. Most of the radiation is absorbed near the absorber wall, and thus causes a hotspot and raises thermal losses. Furthermore, scattered photons will have a higher probability of being scattered out of the absorber and thus lower the optical efficiency. It shall be remembered that the radiation maximum of the solar spectrum is around λ = 500 nm. Figure 2c shows the third situation with a radiation wavelength of λ = 700 nm. At this wavelength, radiation absorption of the Ag nanoparticles is considerably lower than at the absorption maximum, while H${}_{2}$O, being a good IR-absorber, is still highly transmissive. This situation causes a more homogeneous distribution of spectral radiation intensity among the absorber cross section, as can be seen from the right side of the figure for moderate particle volume fractions ($f_{V}$ = 1 $\times \;10^{-5}$). The impact of deviation of $f_{V}$ is again shown on the right side of the figure along the black line. It can be seen that the homogeneous distribution can switch to a radiation maximum in the center for low $f_{V}$ and towards a surface absorber for high $f_{V}$.

The fourth situation (Figure 2d) depicts the case of nearly no absorption by the Ag nanoparticles and high absorption rate of the base fluid which, for example, occurs at λ = 1550 nm. The resulting distribution of spectral intensity is similar to the case of λ = 400 nm, as most of the radiation is absorbed close to the absorber surface (note again the log-scale). The major difference can however be seen looking at the variation of $f_{V}$, again shown at the right side of the figure along the black line. Since the absorption of Ag particles is very low at this wavelength, the distribution is hardly affected by a variation of $f_{V}$.

These findings are of great importance when designing a suitable absorber. A good way of optimization would be to first adapt the absorber diameter in a way that no photons pass the absorber in the IR wavelength range, where the absorption of the base fluid dominates. The particle volume fraction $f_{V}$ can be adapted in a second step to adjust the short wave absorption so that the radiation can penetrate deeper into the absorber and thus lower thermal losses.

**Figure 3:**
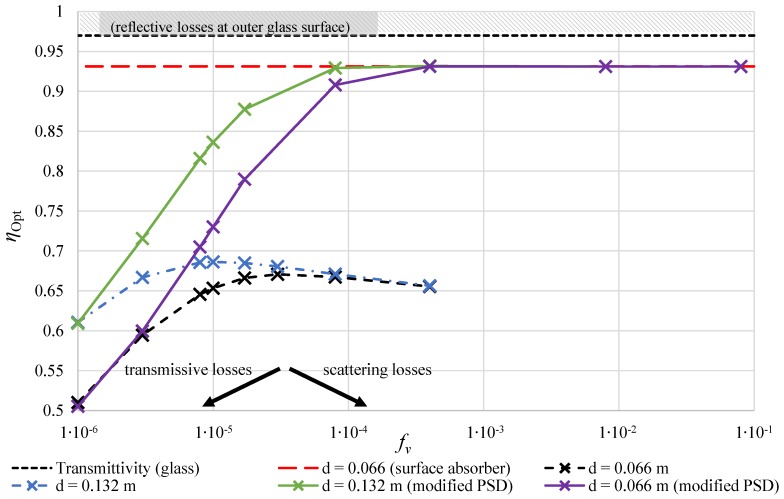
At some point, the benefit of better absorption due to an increase of $f_{V}$ reverts and optical losses due to scattering deteriorate the optical efficiency (dashed lines). For very small particle size distributions (solid lines), less radiation is scattered so that very high absorption rates can be achieved.

To summarize our findings, the optical efficiencies of the absorbers were evaluated, as shown in Figure 3. Since the volumetric absorber is still a concept, several assumptions were made. The outer glass surface of the vacuum insulation causes reflective losses, so that τ = 97% of the incident radiation reaches the absorber [31]. This loss is assumed for the volumetric as well as for the surface absorber, so that the maximum efficiency that the calculations can yield is 97%. This value is hatched in the figure. The only further loss that is considered for the surface absorber in our calculations is the surface absorptivity of 96% [31]. The connection of these losses is an optical efficiency of $\eta_{opt}$ = 0.931, which is henceforth assumed as the surface absorber efficiency. The value is shown as a red dashed line in Figure 3.

For the volumetric absorbers, the radiation has to travel through another glass surface with a transmittance τ. The optical efficiency is defined as follows:

(2) $$\eta_{opt}=\tau \cdot \tau \cdot \frac{\mathrm{absorbed}\;\mathrm{radiation}}{\mathrm{absorbed}+\mathrm{transmitted}+\mathrm{scattered}\;\mathrm{radiation}}.$$

For the simulations, two different PSDs were used. For the measured PSD, from the validation, the efficiency is shown as a black dashed line in Figure 3 for the absorber with an inner diameter of *d* = 0.066 m. It can be seen that fewer photons are transmitted through the absorber with increasing particle fraction $f_{V}$ so that the efficiency increases. At approximately $f_{V}$ = 8 $\times \;10^{-5}$, this trend reverses. The reason is that the average penetration depth gets smaller so that photons get scattered out of the absorber tube. Thus, further increase of the particle fraction causes the absorber efficiency to decline. It is argued above that a larger absorber diameter with a lower particle fraction can raise the absorber efficiency. This can be seen from the blue dashed line in Figure 3. The calculations were repeated with a doubled inner absorber diameter of *d* = 0.132 m. Compared to the smaller diameter, the efficiency is higher at small particle fractions $f_{V}$. This is due to the longer optical path length and, thus, fewer photons being transmitted through the absorber. However, with increasing particle fraction $f_{V}$ the same effect as for the smaller absorber can be observed. At a particle fraction of approximately $f_{V}$ = 1 $\times \;10^{-5}$, the loss due to scattering causes a decrease of the optical efficiency. The best efficiency of such system is around $\eta_{opt}$ = 0.7 which is significantly lower than that of the surface absorber. It can be concluded that scattering is a very important issue and must not be neglected in the analysis of volumetric solar absorber systems.

This decay of efficiency due to scattering is discussed in the following. Therefore, a modified PSD is assumed with very small particles and a very narrow diameter range from 10 nm to 26 nm which yields size parameters between *x* = 0.005 and *x* = 0.2 and thus could be described using the Rayleigh approximation, with which scattering intensity is proportional to $1/\lambda^{4}$ [32]. Since Mie theory covers all cases of size parameters, this setup was calculated using the theory described in the following section. It can be seen in Figure 3 that for the smaller absorber (purple solid line) the efficiency is the same as for the measured PSD at very low volume fractions $f_{V}$. This is due to the high penetration depth and the fact that radiative losses are mainly due to transmission of light through the absorber. At increasing $f_{V}$, an important difference can be observed. Less deterioration of the efficiency to scattering can be observed in comparison to the actual PSD as no large particles are inside the absorber. In fact, the absorber efficiency of this theoretical setup reaches very high values that are equal to the surface absorber efficiency from $f_{V}\geq$ 4 $\times \;10^{-4}$ with a peak value of $\eta_{opt}$ = 0.931. With further increase, the efficiency remains at a high level with a slight decrease. The trend is the same for the larger absorber (green solid line). Similar to the measured PSD, the efficiency is at a higher level at small $f_{V}$ because of less transmission due to the higher optical path length. It must be said that very high particle fractions ($f_{V}>$ 4 $\times \;10^{-4}$) are shown for an analysis of the scattering only. It must be considered that at very high particle fractions nanofluids become very viscous and usage as a working fluid increases pumping power. In addition, it is questionable whether such nanofluids satisfy the assumption of independent scattering [30].

### 2.2. Discussion of the Temperature Field

The absorbed radiation causes an increase of enthalpy in the working fluid that is available as usable heat for a subsequent process. The temperature field that is produced by the inhomogeneous absorption of radiative energy is discussed below.

**Figure 4:**
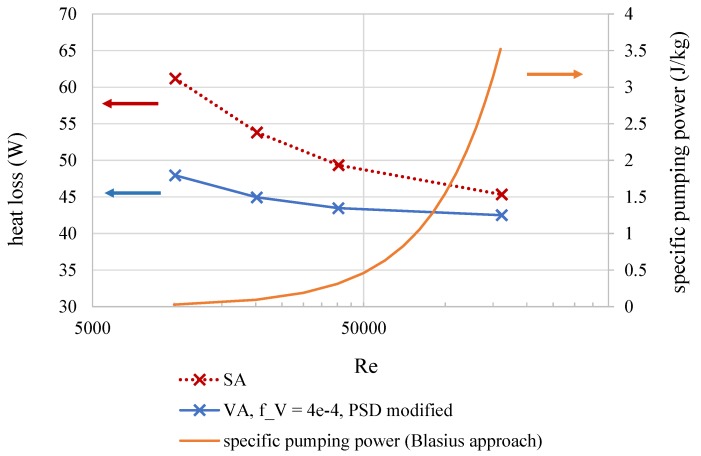
High Reynolds numbers are required in the surface absorber to lower the heat loss. This dependency is weaker in volumetric absorber systems. Additionally, the specific pumping power is shown using the Blasius approach for the pressure loss coefficient. Flows at lower Reynolds numbers require significantly less pumping power.

The first aspect to be considered is the influence of flow rate on the thermal losses from the absorber surface along a section of *l* = 5 m length. For that, the surface absorber was directly compared with a volumetric absorber at different mass flow rates, which is shown in Figure 4. For the case of the surface absorber, the heat flux is absorbed at the outer surface and must be transported into the fluid by forced convection. The highest Reynolds number (Re = 1.6 $\times \;10^{5}$) depicts a mass flow rate of $\dot{m}$ = 8 kg/s at which surface absorbers commonly operate [16]. At such Reynolds numbers, the heat transfer rate is so high that the absorber tube shows a lower temperature and thus lower thermal losses. It can easily be seen that a decrease of flow velocity results in a higher heat loss. Regarding the pumping power, elevated Reynolds numbers have a negative impact. The orange line in Figure 4 shows the mass flow specific pumping power for the flow. The function has been calculated using the Blasius approach for the pressure loss. It can be seen that higher Reynolds numbers cause higher operational costs due to an increased energy demand for pumping.

The situation for the volumetric absorber is different because the heat is released inside the fluid volume. However, the thermal losses slightly decrease with the Reynolds number. The reason for this behavior can be found in Figure 2a: Most of the radiation is transformed into enthalpy close to the absorber wall, where the average flow velocity is lower than in the center of the absorber. This results in a temperature rise towards the absorber wall. A higher Reynolds number, and thus increased turbulence, will enhance the heat transfer towards the wall as well as improve the equalization of temperature among the absorber cross section. This results in a slightly lower heat loss at higher Reynolds numbers. At very low Reynolds numbers, the onset of a layering of the temperature caused by natural convection effects was observed. This effect is unwanted as it would result in higher temperatures at the top of the absorber, thus increasing thermal losses. It must be taken into account that the data in Figure 4 is calculated at a constant absorber length of *l* = 5 m. Hence, lower mass flows reach higher total temperatures at the outlet, which of course has an impact on the heat loss.

**Figure 5:**
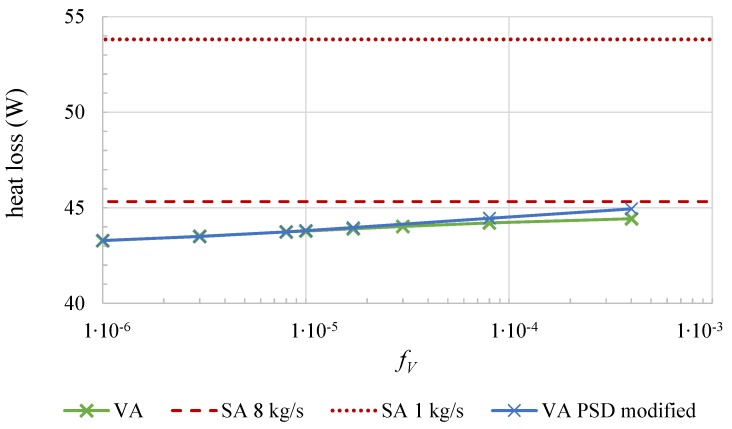
The heat loss is shown as a function of the nanoparticle volume fraction $f_{V}$. The red dashed and dotted lines show the heat loss for the SA, whereas the solid lines describe the VA at two different mass flow rates, respectively.

Figure 5 shows the heat loss over the same section as before for different volume fractions $f_{V}$. The heat loss for the surface absorber at two different mass flow rates are given as constant lines for comparison. It can be seen that the heat loss of the volumetric receiver, which is shown for $\dot{m}$ = 1 kg/s, increases with $f_{V}$, however remains at a lower level even than the SA at $\dot{m}$ = 8 kg/s. It must be considered that the higher mass flow causes a lower average outlet temperature as the thermal capacity flow increases. The difference between the thermal losses of the VA with the actual and the modified PSD is low.

**Figure 6:**
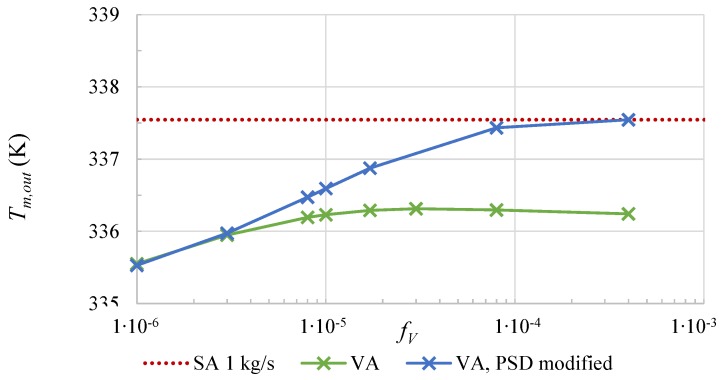
The average fluid temperature $T_{m,out}$ at the outlet as a function of the volume fraction of the VA (solid lines) at a mass flow rate of $\dot{m}$ = 1 kg/s. For comparison, the average fluid temperature of the SA is shown as red dotted line.

Figure 6 shows the increase of the average temperature over the same section of l=5m for a constant mass flow rate as a function of the particle volume fraction $f_{V}$. Due to the lower optical efficiency, the VA (green line) cannot reach the same temperature raise between inlet and outlet as the SA (red line). The effect of a decrease of the optical efficiency at higher $f_{V}$ (compare Figure 3) also causes a lower outlet temperature at rising $f_{V}$. The scattering losses are significantly lower for the modified particle size distribution (blue line). The solar thermal absorber can reach the same average temperatures as the surface absorber (red dotted line) with this modification.

**Figure 7:**
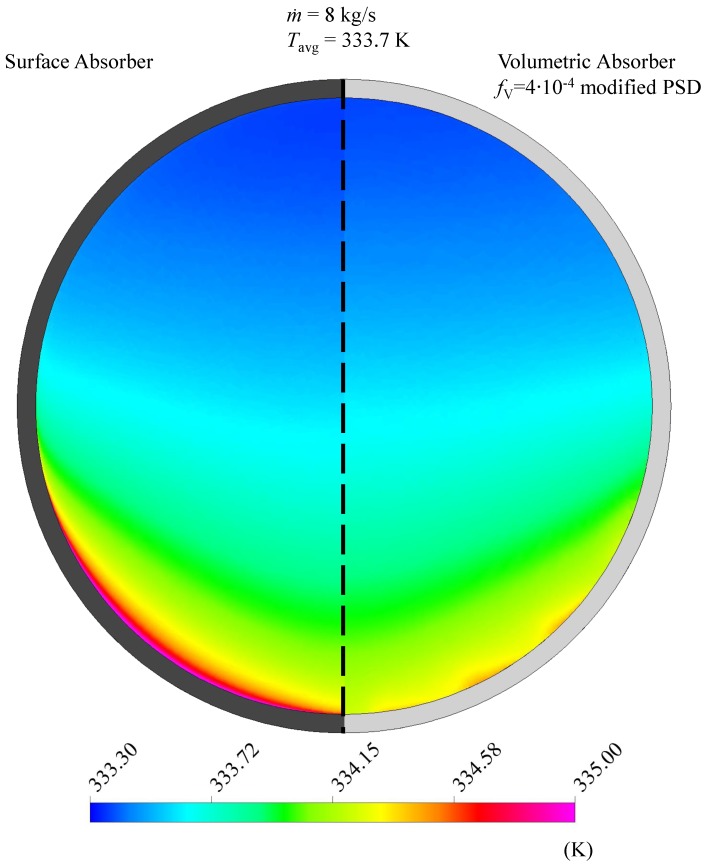
At the same average temperature of $T_{avg}$ = 335.29 K, the temperature distribution is less homogeneous in the surface absorber (left) than in the volumetric absorber (right).

To compare the temperature field among the entire absorber cross section, Figure 7 exemplarily shows the surface absorber and the volumetric absorber with $f_{V}$ = 4 $\times \;10^{-4}$ modified PSD. Both cases have the same optical efficiency, thus absorbing the same amount of energy at the flow length of *l* = 4 m where the cross sections are shown. As the specific heat capacity hardly deviates for small $f_{V}$, the average temperature increase compared to the inlet temperature of *T* = 333.15 K is similar for both cases. However, it can be seen in Figure 7 that the absorber design causes a different temperature distribution: Whereas high temperatures occur in the vicinity of the wall in the surface absorber, the fluid is heated more homogeneously in the volumetric absorber.

## 3. Numerical Methods

Numerical calculations shown in this contribution are carried out with Ansys CFX, where the general continuity equation

(3) $$\frac{\partial \rho }{\partial t}+\nabla \cdot \left(\rho \cdot U\right)=0$$

with the velocity vector *U*, density ρ and the total energy equation

(4) $$\frac{\partial (\rho \cdot h_{tot})}{\partial t}-\frac{\partial p}{\partial t}+\nabla \cdot \left(\rho \cdot U\cdot h_{tot}\right)=\nabla \cdot \left(\lambda_{th}\cdot \nabla T\right)+\nabla \cdot \left(U\cdot \tau \right)+U\cdot S_{M}+S_{E}$$

with the total enthalpy $h_{tot}$, stress tensor τ, thermal conductivity $\lambda_{th}$, momentum source $S_{M}$ and energy source $S_{E}$ are solved. The following sections give detailed information about the numerical methods used and assumptions that are made.

### 3.1. Geometrical Description

Design considerations of the volumetric nanofluid absorber are speculative since no real system has been built yet. Hence, geometrical considerations follow existing systems from concentrating solar thermal power plants, e.g., the established PTR70 receivers by SCHOTT. Its absorber tube’s length of *l* = 5 m inner diameter of *d* = 66 mm is adopted as flow diameter for the following calculations, as well as its wall thickness of 2 mm. Information about this system is taken from [16,31]. The wall material for the volumetric absorber is assumed as glass with a thermal conductivity of $\lambda_{th}$ = 1.2 W/m/K, and for the surface absorber as DIN 1.4541 steel [31] with $\lambda_{th}$ = 16 W/m/K ( [33]).

**Figure 8:**
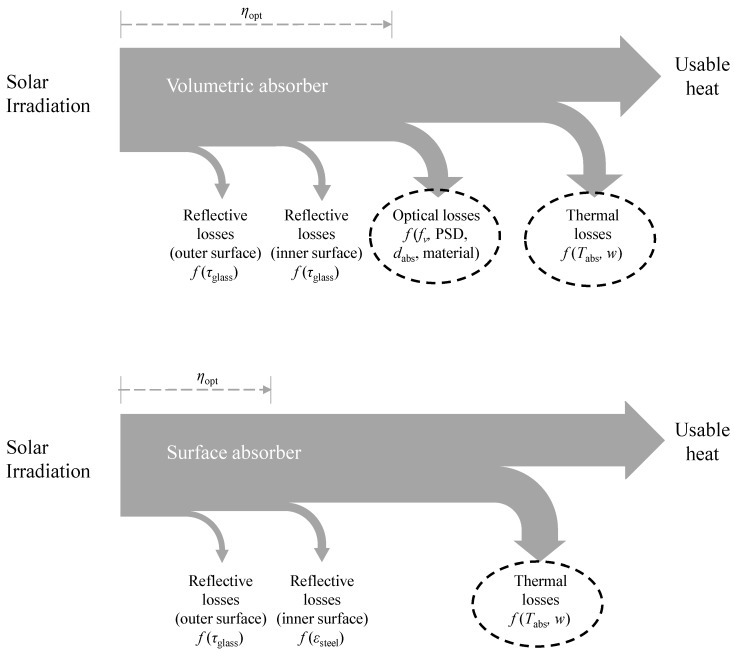
The energy flow chart shows the main difference between the surface absorber and the volumetric absorber system.

The energy flows for the volumetric absorber as well as for the surface absorber are shown in a schematic manner in Figure 8. It can be seen for the volumetric absorber that the radiation passes two layers of glass (compare Figure 1) before entering the absorber fluid, each layer causing reflective losses as explained later. The space between the two glass layers contains a vacuum to reduce convective heat transfer away from the absorber. How much usable heat the absorber provides is determined by the optical and thermal losses. The situation is slightly different for the surface absorber. Radiation passes the outer glass surface and is subsequently absorbed by the blackened surface. Both produce certain reflective losses, as discussed below. However, it shall be revealed that the difference of the reflective losses due to the two glass layers (τ·τ = 0.9404) and the reflective losses in the surface absorber (τ·α = 0.9312) are small. The judgment whether these mechanisms could be improved for volumetric absorbers by addition of special coatings or one-way mirrors go beyond the scope of this contribution. The use of selective coatings was omitted in the calculations. Though these coatings are already applied in many existing plants [16,31], a comparison with a fictitious volumetric absorber would be difficult. However, selective coatings for volumetric nanofluid absorbers are already being discussed in the literature (e.g., [34,35]). Thus, it can be concluded in Figure 8 that the margin in which the optical performance of the absorber can be improved is small. Which absorber provides more usable heat is hence mainly determined by the ratio of optical losses and thermal losses in the volumetric absorber and the thermal losses in the surface absorber.

### 3.2. Flow Field

Burkholder and Kutscher published an efficiency analysis of the SCHOTT PTR70 receiver [16] in which a flow rate of $\dot{m}$ = 8 kg/s is assumed. With the inner diameter of *d* = 66 mm, this results in a turbulent flow. In our contribution, temperature and flow fields are examined at different flow rates, with all of them being in the turbulent regime. As demonstrated below, velocities that are too low result in thermal layering of the flow, which is undesirable due to increased heat losses. The k-ω-model is used for turbulence modeling. Buoyant forces are calculated using the Boussinesq approximation. The no-slip condition is set for the inner tube surface. Calculations are performed in a stationary state. Prior to radiation and thermal simulations, the turbulent flow field for an isothermal case was calculated and used as inlet boundary condition to obtain a hydrodynamically developed flow. The structured mesh consists of 3.9 million elements.

### 3.3. Nanofluid Modeling

The discussion about thermophysical properties of nanofluids in literature is very lively [5,12]. However, volume fractions of the nanofluids considered in this contributions are very low ($f_{V}$ = 1 $\times \;10^{-6}$, ..., 4 $\times \;10^{-4}$). Since the volume fraction is usually used as a proportionality factor in property correlations, consider, e.g., the Maxwell and the effective medium theory for the effective thermal conductivity, the choice of a certain correlation is less important than in calculations with higher $f_{V}$. In fact, thermophysical properties of the nanofluids used hardly differ from those of pure DI water, which is used as base fluid (bf) [12]. Hence, simple correlations for thermophysical properties of the nanofluids are used in our numerical calculations: The effective density is calculated using the well-known correlation by Pak and Cho ($\rho_{H_{2}O}$ = 997.3 kg/m${}^{3}$, $\rho_{eff}$ = 1001.1 kg/m${}^{3}$) [2]

(5) $$\rho_{eff}=\rho_{p}\cdot f_{V}+\rho_{bf}\cdot \left(1-f_{V}\right),$$

whereas the approach found by Xuan and Roetzel is used for the specific heat ($c_{p,H_{2}O}$ = 4181.8 J/(kgK), $c_{p,eff}$ = 4164.3 J/(kgK)) [4]

(6) $$c_{p,eff}=\frac{f_{V}\cdot \rho_{np}\cdot c_{p,np}+\left(1-f_{V}\right)\cdot \rho_{bf}\cdot c_{p,bf}}{\rho_{nf}}.$$

Both models has been validated and discussed for similar volume fractions in prior studies [12]. Furthermore, the thermal conductivity is calculated with the Maxwell equation ($\lambda_{H_{2}O}$ = 0.605 W/(mK), $\lambda_{eff}$ = 0.606 W/(mK)) [36]

(7) $$\lambda_{nf}=\lambda_{bf}\frac{\lambda_{np}+2\cdot \lambda_{bf}+2\cdot \left(\lambda_{np}-\lambda_{bf}\right)\cdot f_{V}}{\lambda_{np}+2\cdot \lambda_{bf}-\left(\lambda_{np}-\lambda_{bf}\right)\cdot f_{V}}$$

and the viscosity with the Einstein approach ($\eta_{H_{2}O}$ = 9.544 $\times \;10^{-4}$ Pa s, $\eta_{eff,Ein}$ = 9.554 $\times \;10^{-4}$ Pa s) [37,38]

(8) $$\eta_{eff}=\eta_{bf}\cdot \left(1+2.5\cdot f_{V}\right).$$

The effective nanofluid viscosity calculated with the Einstein approach shown above, compared with the effective viscosity using, e.g., the Batchelor approach shows again that the models hardly differ in this range of volume fraction ($\eta_{eff,Bat}$ = 9.550 $\times \;10^{-4}$ Pa s). Further details about the simulated Ag-H${}_{2}$O nanofluid, such as particle size distribution (PSD) and zeta potential, are given in the validation section.

### 3.4. Thermal Modeling

Nanofluids have not yet been used in high temperature or high pressure applications for longer periods. Without doubt this is an urgent need for research which still impedes nanofluids from large-scale use. For this reason, the penetration of radiation and the resulting temperature profiles are analyzed at a low inlet temperature of 333.15 K and atmospheric pressure. This is a limitation of our calculations with regard to a usage in the field, as thermal losses are at a low level at these temperatures. However, more basic research about nanofluid properties is necessary for a decent extrapolation into regions of higher temperature and pressure. However, a lot can be learned about the penetration of radiation into the fluid and about heat transfer mechanisms in a volumetric absorber even at lower temperatures.

The absorber surface is thermally connected to the surrounding with a heat flux

(9) $$\dot{Q}=0.141\cdot \vartheta_{\mathrm{abs}}+\left(6.48\;\times \;10^{-9}\right)\cdot \vartheta_{\mathrm{abs}}^{4},$$

with $\vartheta_{\mathrm{abs}}$ being the absorber temperature in ${}^{\circ }$C. The correlation is found by Burkholder and Kutscher [16] for the SCHOTT PTR70 absorber. However, it must be said that the authors developed this correlation from experiments between 100 ${}^{\circ }$C and 500 ${}^{\circ }$C, so that the range is extrapolated downwards. For better comparison, this approach is used to calculate thermal losses of both the SA system and the VA system, as the same glass insulation can be assumed.

### 3.5. Radiation Modeling

It has been described above that the principle of volumetric absorption aims at moving the temperature maximum into the liquid volume. This shall be achieved by radiation that penetrates into the nanofluid where radiative energy is transformed into enthalpy. This concept should not be discussed without a spectral sensitive analysis of the system. To illustrate this fact, the expected function of radiation intensity over the penetration depth in the volumetric absorber tube is discussed: In absence of an absorbing or scattering medium the radiation intensity should increase towards the center of the tube, as a constant radiative energy flux focuses on a decreasing area. However, the incident radiation varying over the circumference of the absorber tube is an aggravating factor. Considering absorption and scattering along a straight line in an absorbing and scattering medium an exponential decay of radiation intensity following the Beer-Lambert law is expected. Thus, it is very difficult to predict the radiation intensity, being a superposition of the effects named above. The exponential decay is determined by the absorption and scattering coefficients which in turn are a function of the radiation wavelength. Hence, such simulation must be conducted with a numeric code that considers spectral dependencies. This means that the penetration depth for different wavelengths can be evaluated which will provide more information about a possible absorber design and suitable nanofluids.

**Figure 9:**
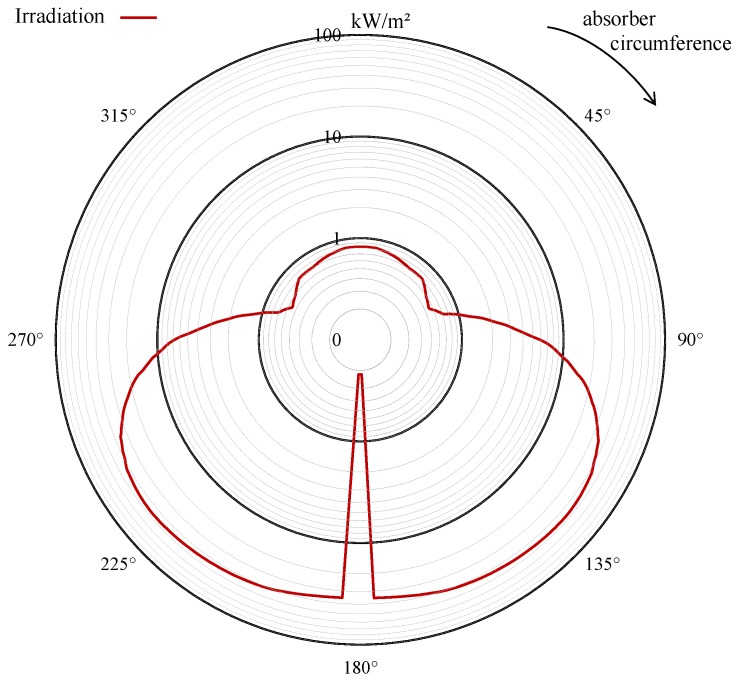
Polar plot of solar irradiation over the absorber circumference in log-scale. The solar collector causes high radiation fluxes from the bottom of the absorber while unconcentrated solar radiation is incident on the top of the absorber.

Figure 9 shows the irradiation boundary condition considered for the solar thermal absorber. The data from a publication by Wirz [39] are modified by assuming axial symmetry. Furthermore, the authors data on the absorbers transmissivity and absorptivity are replaced with values from [31]. The spectral distribution of the irradiation flux is calculated using the NREL ASTM G173-03 Reference Spectra (Direct + Circumsolar). Figure 9 shows that the unfocused insolation irradiates the top side of the absorber. The bottom of the absorber receives the radiation concentrated by the collector system which is some orders of magnitude stronger. At the center of the absorber at the bottom, the shadow of the absorber tube itself becomes visible, which causes a strong decrease of irradiation at this point. In general, it can be seen that the insolation on the absorber surface is very inhomogeneous.

The Monte Carlo model of Ansys CFX is used for radiation modeling to solve the radiative transfer equation. To allow for spectral differences of the absorption and scattering coefficient, as well as for an adequate simulation of the intensity distribution over the solar spectrum, the Monte Carlo model is used in combination with the multi band option. The calculation was discretizied in 104 spectral bands, covering the solar spectrum from λ = 0.3 μm to λ = 4 μm. The spectrum of the Philips 13117 halogen spots that are used in the validation measurements is discretized into 80 spectral bands. Data of the radiation intensity distribution of the solar spectrum are taken from the official NREL ASTM G173 Reference Spectra, data for the Philips 13117 Halogen spots stem from [40].

Spectral absorption and scattering coefficients of the Ag nanofluid are calculated using the Mie theory. Some authors claim that simplified calculations using the Rayleigh scattering approach is sufficient or that scattering can be neglected due to the small size of nanoparticles. As shown in the results section, this assumption does not necessarily hold true for real nanofluids and can cause significant errors in the simulation of the radiation and temperature fields (e.g., [41]).

The following calculations of the radiative properties are performed using a mathematical derivation that can be found in [42,43]. For each size parameter $x=\pi 2an_{M}/\lambda$, with the particle radius *a*, the refractive index of the medium $n_{M}$ and the vacuum wavelength of incident radiation λ, the Mie parameters $a_{n}$ and $b_{n}$ can be calculated:

(10) $$\begin{array}{cc} a_{n} & =\frac{\psi_{n}\left(x\right)\psi_{n}^{\prime }\left(mx\right)-m\psi_{n}^{\prime }\left(x\right)\psi_{n}\left(mx\right)}{\xi_{n}\left(x\right)\psi_{n}^{\prime }\left(mx\right)-m\xi_{n}^{\prime }\left(x\right)\psi_{n}\left(mx\right)} \end{array}$$

(11) $$\begin{array}{cc} b_{n} & =\frac{m\psi_{n}\left(x\right)\psi_{n}^{\prime }\left(mx\right)-\psi_{n}^{\prime }\left(x\right)\psi_{n}\left(mx\right)}{m\xi_{n}\left(x\right)\psi_{n}^{\prime }\left(mx\right)-\xi_{n}^{\prime }\left(x\right)\psi_{n}\left(mx\right)}. \end{array}$$

In Equations (10) and (11), $\Psi_{n}\left(x\right)$ and $\xi_{n}\left(x\right)$ are the Riccati–Bessel and Riccati–Hankel functions with the according derivatives. The parameters $c_{n}$ and $d_{n}$ for the internal field are omitted and can be found in [43]. The parameters $a_{n}$ and $b_{n}$ can be used to calculate the extinction cross section $C_{\mathrm{ext}}$, scattering cross section $C_{\mathrm{sca}}$ and absorption cross section $C_{\mathrm{abs}}$:

(12) Cext=2πkM2∑n=1∞(2n+1)Re(an+bn)

(13) $$\begin{array}{cc} C_{\mathrm{sca}} & =\frac{2\pi }{k_{M}^{2}}\sum_{n=1}^{\infty }\left(2n+1\right)(|a_{n}{|}^{2}+|b_{n}{|}^{2}) \end{array}$$

(14) $$\begin{array}{cc} C_{\mathrm{ext}} & =C_{\mathrm{abs}}+C_{\mathrm{sca}}, \end{array}$$

which have the dimension of an area. The physical meaning of the summation parameter *n* in Equations (12)–(14) are plasmonic oscillation modes that are excited in the particles. How many of these modes need to be considered for a precise calculation at moderate calculation times was suggested by Wiscombe [44]:

(15) $$n_{\max}=\left\{\begin{array}{ll} \mathrm{integer}\left(x+4x^{1/3}+1\right) & f\ddot{u}r\;x\leq 8\\ \mathrm{integer}\left(x+4,05x^{1/3}+2\right) & f\ddot{u}r\;8<x<4200\\ \mathrm{integer}\left(x+4x^{1/3}+2\right) & f\ddot{u}r\;4200\leq x \end{array}\right.$$

Using $N_{V}$ as the amount of particles per volume, the above equations can finally be used to calculate the extinction coefficient β, the scattering coefficient $\sigma_{s}$ and the absorption coefficient κ:

(16) $$\begin{array}{cc} \beta & =N_{V}C_{\mathrm{ext}} \end{array}$$

(17) $$\begin{array}{cc} \sigma_{s} & =N_{V}C_{\mathrm{sca}} \end{array}$$

(18) $$\begin{array}{cc} \kappa & =N_{V}C_{\mathrm{abs}}. \end{array}$$

**Figure 10:**
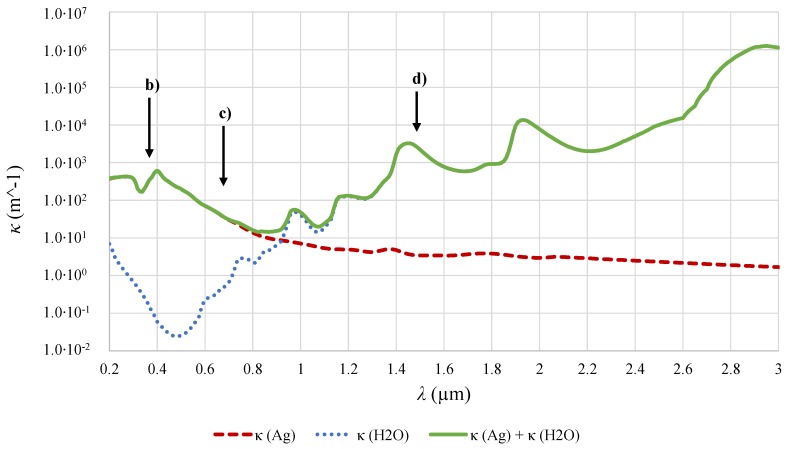
Absorption coefficient κ as a function of wavelength λ. Absorption by nanoparticles dominates in the UV/Vis spectral region, whereas it is almost negligible in the IR spectral range where the influence of the base fluid predominates.

These parameters can be implemented into the numerical calculations. Note that property data for the complex index of refraction are needed for the calculation in Equations (10) and (11). For the Ag nanoparticles, data from Babar and Weaver are used [45]. Data for the base fluid H${}_{2}$O are taken from Hale and Querry [46]. For better comprehension of the results section, the absorption coefficients of H${}_{2}$O, Ag nanoparticles as well as the superposition of both functions are shown in Figure 10. The letters (b), (c) and (d) refer to selected wavelengths that has been discussed in more detail before (see Figure 2). It can be seen in the figure that radiation with smaller wavelength (up to 700 nm) is predominantly absorbed by particles, whereas absorption by the base fluid H${}_{2}$O dominates in the infrared region of the spectrum. More information about measured and calculated absorption and scattering coefficients can be seen in [29].

Though above calculations look straightforward at first sight, there are some important issues to be considered. First, the transformation from the optical cross section described in Equations (17) and (18) assumes a constant particle size, which in actual nanofluids is too much of a simplification. One way to overcome this issue is described in [47], where particle size distribution functions are implemented in the calculations. However, measured size distributions, such as shown in Figure 13, can vary significantly from the suggested Gamma, Weibull or Delta distribution functions so that such an assumption can fail. In our calculations, the Mie parameters are determined for any particle size and wavelength, including the spectral material properties. To obtain spectral dependent optical parameters, we then iteratively *fill* a unit volume with single particles according to the quantity size distribution function until $f_{V}$ is reached. This method can assume any real distribution function, however it demands more calculation time.

The next issue addresses the implementation of the calculated scattering coefficient from Equation (17) in the numerical calculations. The way scattering is handled in CFX will yield incorrect results, as by default an isotropic scattering phase function is assumed. In general, a scattering phase function expresses the probability of a photon being scattered into a certain angle Θ (e.g., explained in [32]), with Φ(Θ)=1 describing the case of isotropic scattering. This means with the assumption of isotropic scattering photons are equally scattered into each direction.

**Figure 11:**
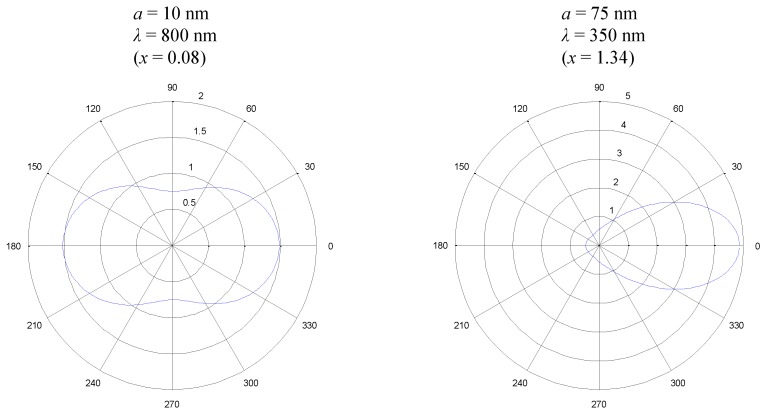
The scattering phase function for spherical particles is, besides material properties, a function of particle size *a* and wavelength λ of the incident light (irradiation from the left side of the image).

The phase function calculated for two different size parameters *x* are shown in Figure 11. The left hand side represents a very small particle from the size distribution with *a* = 10 nm radius being irradiated by IR radiation with λ = 800 nm. It can be seen from the phase function that the incident radiation coming from the left side is equally scattered forward and backwards, which is depicted by the Rayleigh scattering regime. The situation on the right hand side of Figure 11 is different: Light in the UV wavelength of λ = 350 nm is incident on a larger spherical particle with *a* = 75 nm. The phase function shows that most of the light is scattered into a forward direction. Both cases, as well as several more, must be considered in our calculations because the assumption of isotropic scattering will yield incorrect results. Especially, short wave solar radiation would suffer too strong backscattering by larger particles. This would result in lower penetration depths of the radiation into the nanofluid as well as a lower absorber efficiency due to photons leaving the domain because of overestimated backscattering. To take such effects into account, Ansys CFX allows for implementation of a linear anisotropic phase function. However, calculations quickly show that a linear anisotropic approach is not enough to account for the strong forward scattering and only yields slightly improved results.

Therefore, an approach is used that evolves from a method suggested by Modest and Azad some years ago [48]. The authors argued that situations in which great parts of the radiation is scattered in a forward direction can be simplified by first assuming an isotropic scattering behavior for the scattering angles that do not deviate from such behavior too strongly. The forward scattering that exceeds the isotropic scattering phase function can then be considered as transmission. From the energetic perspective, this can be justified as it makes no difference in the radiative transfer equation whether a photon is transmitted or scattered in the forward direction. However, it must be said that this is not necessarily true for the entropy of the radiation as the polarization of light might be affected by the scattering event. The transmitted portion of light can be accounted for by calculating a spectral dependent, modified scattering coefficient $\sigma_{s}^{*}$ which is described in [48]. The calculation is performed iteratively by finding a modified linear anisotropic scattering phase function $\Phi^{*}\left(\Theta \right)$ which satisfies the equations:

(19) $$\delta_{f}=\frac{1}{2}\int_{\mu_{f}}^{1}\left[\Phi \left(\mu \right)-\frac{\sigma^{*}}{\sigma }\Phi^{*}\left(\mu \right)\right]$$

and

(20) $$\sigma^{*}=\sigma \left(1-\delta_{f}-\delta_{b}\right).$$

Modest and Azad described that a forward scattering angle $\Theta_{f}$ (i.e., $\mu_{f}=\cos\left(\Theta_{f}\right)$) needs to be defined in which scattered radiation exceeding the isotropic assumption is assumed as transmitted light [48]. In our calculation, this angle is defined as the point where forward and backward scattering (compare Figure 11) are equal but 90${}^{\circ }$ at most. This scattering angle is calculated for every combination of *a* and λ. Besides the correction term $\delta_{f}$ for forward scattered radiation (Equation (19)), a similar term $\delta_{b}$ is included for backscattering in Equation (20). Modest and Azad suggested neglecting this term to improve simplicity of the method [48]. This can be justified by taking into account that during our study numerous combinations of radiation wavelength, particle size and refractive indices were found where forward scattering dominates strongly (compare, e.g., Figure 11), whereas no comparable cases of backscattering could be found.

The calculations yield a modified scattering coefficient $\sigma_{s}^{*}$ being a function of particle size and radiation wavelength. Since CFX can only account for the spectral dependence of $\sigma_{s}^{*}$ the size dependency were suspended by using the particle size distribution for weighting. The result can then be imported into CFX as a spectral dependent scattering coefficient along with the spectral absorption coefficient κ.

Another issue addresses the calculation of the radiation field in Ansys CFX. The Monte Carlo model used in our simulation can consider participating media but cannot satisfactorily account for transparent walls. However, if a photon travels through the whole absorber tube without being absorbed on the way or on the other hand is scattered backwards from the very first nanoparticles, it will leave the absorber domain and hence decrease the optical efficiency. In Ansys CFX, this photon would instead be absorbed by the wall or even be reflected back into the absorber liquid until it will be absorbed in the volume. Both cases would fail to yield correct results and overestimate the absorber performance. To overcome this problem, the simulation was divided into a radiative and a thermal analysis: First, the radiation field inside the absorber was simulated for an isothermal fluid and isothermal walls with an emissivity of 1. This means that any photon being transmitted through the domain or scattered out of the volume will be absorbed by the isothermal domain walls. Subsequently, the absorbed radiative energy per volume was exported for each mesh element and implemented into the thermal simulation as a volumetric source term. This procedure allows for considering photons not being caught inside the absorber and, thus, for calculation of the absorber performance.

For the simulation, a geometry was designed according to the already mentioned Schott PTR70. The mesh consists out of elements, that are homogeneously distributed along the flow direction of the absorber tube. In the cross section, the hexahedral elements are decreasing in their size from the center towards the absorber tube wall. Here, it is important to notice that the precision of the Monte Carlo method not only depends on the refinement of the mesh but also on the number of histories, which is often interpreted as the number of photons, that are simulated. Ansys CFX provides two output values, that describe deviations of the mean value of the radiation intensity on element volumes and surfaces. With the very high number of histories (5 $\times 10^{8}$) and 3.9 million elements, the standard deviations in the calculations were distinctively under the suggested values. To avoid interpolation errors during the implementation of the source term, the mesh of the thermal simulation is identical to the radiative simulation, although it is comparatively fine for this problem.

## 4. Experimental Validation

Experiments were conducted to validate the simulation of radiative heating of nanofluids. Since temperature distributions inside the parabolic absorber tube are difficult to obtain without disturbing the flow field, a simplified experimental setup was designed which is explained below.

### 4.1. Experimental Setup

**Figure 12:**
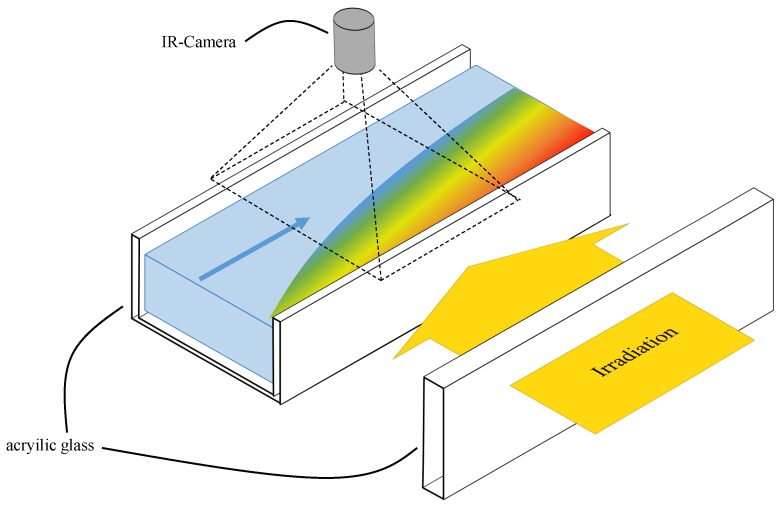
Experimental setup for the validation measurements with IR-camera and open channel.

The absorber fluid flowed inside a rectangular transparent channel that is open at the upper side (see Figure 12). Radiation from 13 Philips 13117 halogen spots penetrated the nanofluid from one side of the channel and is absorbed in the liquid volume. The radiative intensity of the halogen spots after transmission and reflection losses due to the channel wall were measured as a function of the channel length using a Kipp&Zonen CMP10 pyranometer .

The radiative energy was transformed into enthalpy of the nanofluid and causes a temperature distribution inside the liquid. A storage tank for the nanofluid ensured that a stationary state can be achieved for several minutes and a peristaltic pump circulates the liquid. A stirling-cooled Infratec Image IR8300 IR camera was mounted above the channel, looking vertically to the fluid surface from a height of 50 cm and recorded the temperature distribution at the liquids surface. The IR camera has a spectral working range of λ = 2000–5700 nm, a temperature resolution of 20 mK at 30 ${}^{\circ }$C and a measurement accuracy of 1%. The lowest absorption coefficient of the base fluid water in this range is α = 16.5 cm^−1^ at a wavelength of λ = 2200 nm [46]. This value correlates to a depth of 0.6 mm at which incident radiation intensity decreases to a value of 1/e [49]. Hence, the evaluation of the temperature field measured with the IR camera implied the assumption that the change of temperature distribution in vertical direction is negligible within the first ≈ 1 mm of the fluid field. In other words, it was assumed that the emitted radiation from the surface of the liquid is predominant in the signal recorded by the IR camera.

### 4.2. Nanofluid Preparation

**Figure 13:**
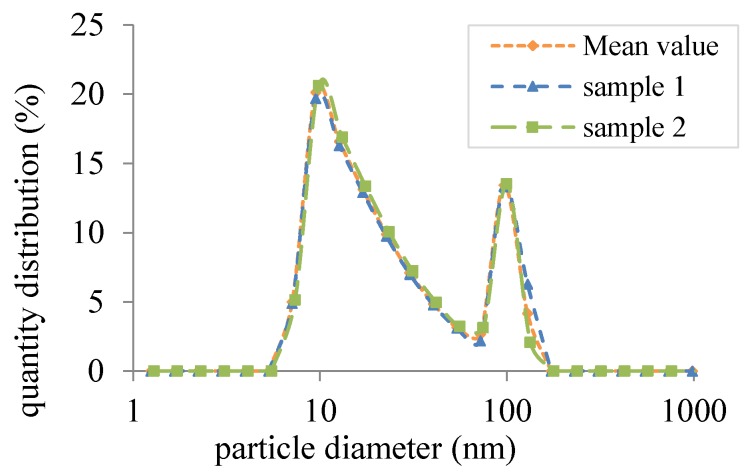
Quantity distribution of the Ag-H${}_{2}$O nanofluid after dispersion.

Besides the experimental setup, the sample preparation is an important issue in nanofluid research [50] to obtain reproducible results. The Ag nanoparticles produced by *Nanotechnologies* with primary particle size 20 nm were weighed with the balance *ABT 120-5DM Kern*. Subsequently, samples with $f_{V}$ = 1.71 $\times \;10^{-5}$ were dispersed in deionized water with an ultrasonic processor (*Hielscher UP100H*) for 45 min in a cooling bath. Since the PSD is considered in the radiation simulation, it was measured using a NANOPHOX measurement system by Sympatec, based on photon cross-correlation spectroscopy (see Figure 13).

**Figure 14:**
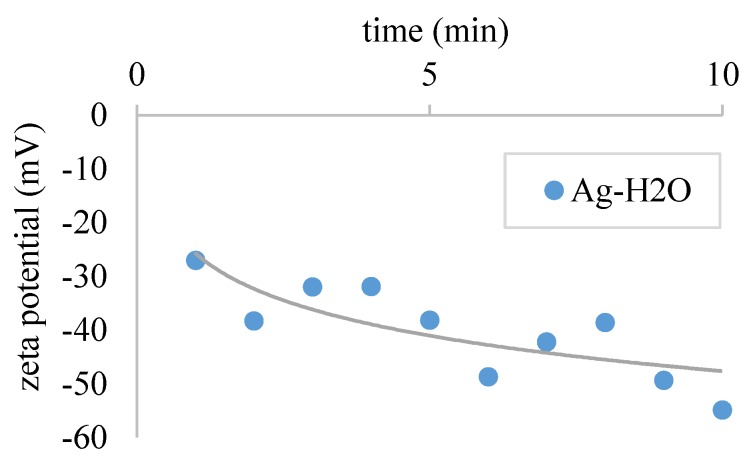
Zetapotential after sonication process. The line is inserted manually to guide the eyes.

The experiments were conducted immediately after the preparation process to avoid errors due to agglomeration and settling effects. To show that the Ag-H${}_{2}$O Nanofluid is stable for some time, the zeta potential was measured for 10 min immediately after the sonication process using a Brookhaven ZetaPals measurement system. Stability can be seen in Figure 14 since the zetapotential exceeds an absolute value of at least 30 mV [51,52].

Measurements of the temperature field were conducted at two different flow rates of 7.7 mL/s and 9.5 mL/s, which correspond to an average flow velocity of 0.84 cm/s and 1.04 cm/s, respectively.

### 4.3. Validation Measurements

**Figure 15:**
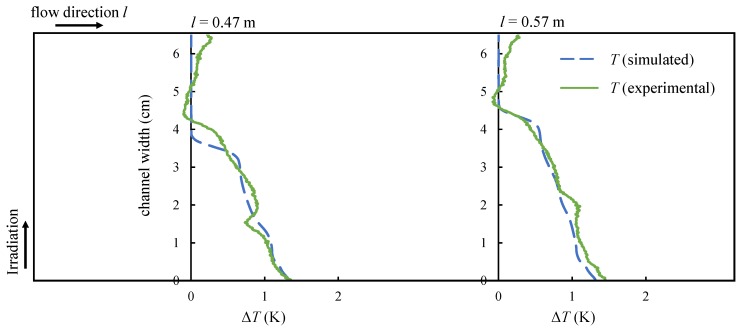
The simulated temperature distribution caused by absorbed radiation matches the experimental values.

To evaluate the measurements, the temperature difference ΔT, being the difference of the local temperature and the free flow temperature at the nanofluid surface orthogonal to the channel wall, is given in Figure 15. Measured (green solid line) and simulated (blue dashed line) values are shown at a channel length of *l* = 0.47 m and *l* = 0.57 m for the lower flow rate of $\dot{m}$ = 7.7 mL/s.

The temperature difference is a consequence of an increase of the nanofluids enthalpy which in turn is caused by absorbed radiation. Hence, it can be seen from the temperature curves that most of the radiation is absorbed in the vicinity of the wall which agrees with the expected behavior from the Beer–Lambert law. The temperature increase decays with growing distance from the wall. The thermal boundary layer becomes thicker with increasing channel length, which can also be seen in Figure 15. The slight temperature increase towards the back wall stems from the channel walls, which had not entirely cooled down from prior experiments. The temperature distribution at the flow surface from the experiments (solid line) is in very good agreement with the simulation results (dashed line), especially regarding the thickness of the thermal boundary layer as well as the fluid temperature in the vicinity of the channel wall.

**Figure 16:**
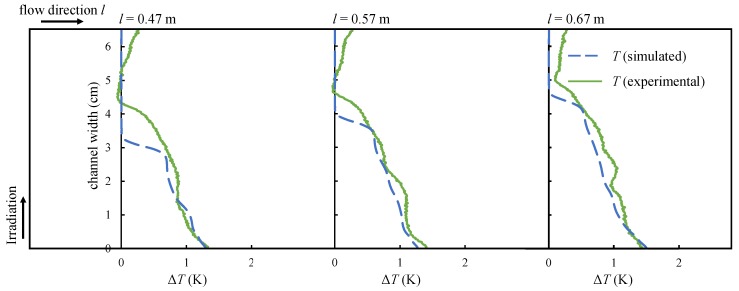
The results for higher flow velocity confirm the good agreement between experiments and simulation.

Figure 16 shows the results for an increased flow rate of $\dot{m}$ = 9.5 mL/s at three different channel sections (*l* = 0.47 m, *l* = 0.57 m and *l* = 0.67 m). The shape of the temperature curve is similar to the case of lower flow velocity. The simulation matches the experimental results though at low channel lengths the thermal boundary layer is calculated to be slightly too thin. The thickness of the thermal boundary layer increases with the channel length and the difference between simulation and experiment becomes smaller. The temperature at the channel wall is calculated with good precision for all situations. Although the experiments have been repeated, the measurement uncertainty is hard to quantify due to varying parameters, such as channel position or flow rate. Additionally, the duration of the experiments proved as a factor too, since impurities in the channel material led to absorbing radiation. Regarding the temperature measurement, the depth of the thermal radiation signal was neglected. Therefore, only temperature differences have been validated, since the measurement accuracy of the IR camera is very high. Besides, the complex structure of the setup allows for further sources of uncertainty such as the measurement of the mass flow as well as the measurement of the radiation intensity of every single halogen lamp especially regarding the angular dependence irradiation on the channel. However, in general, it can be seen that the results seem to agree to a certain rate.

## 5. Conclusions

In this contribution, the radiation and temperature fields inside a volumetric solar thermal absorber were analyzed and compared with a conventional surface absorber. This paper describes how absorption and scattering influences the distribution of radiation within the absorber. It has been shown how optical effects caused by nanofluids can be implemented into numerical software such as Ansys CFX. The advantage of such implementation is that the radiation calculation can be coupled with flow field and thermal simulation. The described model is validated in experimental investigations.

From the evaluation of the simulated radiation fields, the following key findings can be summarized:

- Solely, a high particle fraction $f_{V}$ does not necessarily improve the optical efficiency of a volumetric solar thermal absorber. Whereas transmission lowers the optical performance for small $f_{V}$, scattering deteriorates the performance at high $f_{V}$ (see Figure 3).
- Scattering must not be neglected for solar thermal absorbers with nanofluids, as it has a great impact on the optical efficiency. Especially, realistic particle size distributions cause strong scattering that cannot be accounted for by the assumption of isotropic scattering.
- A very small and narrow particle size distribution must be achieved to obtain an optical efficiency on the same level as surface absorbers.
- The margin to further improve the optical efficiency is small as most of the optical losses occur at the reflective glass surface. It might be possible to improve the surface with selective coatings or one-way mirrors from the inside of the glass tube, which additionally decrease radiation loss due to scattering.

From the thermal analysis, the following can be concluded:

- The assumption that a volumetric absorption of radiation can lower the outside absorber temperature and thus the thermal losses is confirmed.
- The absorber temperatures must be increased for a decent, quantitative analysis of the absorber. At low temperatures, thermal losses are generally decreased so that significant differences between the absorber types are more difficult to identify. However, it could be shown that thermal losses can be reduced in volumetric absorbers. Furthermore, this effect is expected to gain more significance as higher absorber temperatures are achieved. However, basic research about nanofluids at elevated temperatures must be performed.
- The flow velocity must be high in surface absorber systems to cool the absorber wall from the inside and therefore reduce thermal losses to the surroundings. This effect is not necessary for volumetric absorbers. Hence, low thermal losses can be achieved with slower flow velocities, which reduces pressure loss and pumping power.
- For better comparison, the absorber systems was compared with a similar design. However, it must be said that the outer glass cover and the vacuum insulation is not crucial for the volumetric absorber system due to the lower surface temperatures. The glass absorber has a low thermal conductivity (0.8 W/m/K) and thus serves as an insulation material. The omission of the vacuum insulation would mean a severe reduction of production costs. In practical use, a combination seems reasonable: For the cooler parts of the absorber system, the single glass insulation will be sufficient but vacuum insulation can be added for the absorber surfaces with highest temperatures. Note that, due to reflective losses, the omission of the outer glass surface will further increase the optical efficiency of the volumetric absorber.

The energetic efficiency is discussed in this manuscript. It has been shown that, from the consideration of radiative and thermal losses, a volumetric absorber can be a promising approach. A further exergetic discussion of the volumetric absorber compared to the surface absorber could clarify remaining questions.

## Abbreviations

The following abbreviations are used in this manuscript:

VA	Volumetric Absorber
SA	Surface Absorber
PSD	Particle Size Distribution
$f_{V}$	Volume fraction
